# Devemos “Ajustar” Nossa Abordagem à Doença Arterial Coronariana?

**DOI:** 10.36660/abc.20220572

**Published:** 2022-08-25

**Authors:** Andres Felipe Valencia Rendón

**Affiliations:** 1 Universidade Federal do Rio de Janeiro Rio de Janeiro RJ Brasil Universidade Federal do Rio de Janeiro, Rio de Janeiro, RJ – Brasil

**Keywords:** Doença Arterial Coronariana, Aterosclerose, Doenças Cardiovasculares/mortalidade, Biomarcadores, Tecnologia Médica/tendências

Apesar de nossos grandes avanços na tecnologia médica, as Doenças Cardiovasculares continuam sendo a principal causa de morte em todo o mundo. A Doença Arterial Coronariana (DAC) é um dos destaques.^1^

Acredita-se que a primeira correlação entre fluxo coronário e angina pectoris tenha sido feita por William Heberden em 1768, mas suas observações anatômicas só foram descritas em 1829, quando Jean Lobstein introduziu o termo Arteriosclerose, relatando uma formação anormal nas paredes arteriais.^2^ Mais tarde, em 1856, Rudolph Virchow sugeriu um mecanismo de lesão da parede arterial que poderia explicar esses achados de um composto de patologia celular.^3^ No entanto, foi apenas até o século 20, no final dos anos 70, que a associação de dano endotelial-estresse de cisalhamento da parede-proliferação de músculo liso-trombogênese foi elucidada pela primeira vez na “Resposta à Hipótese de Lesão” de Ross et al..^4^

A cardiologia moderna evoluiu exponencialmente desde essas primeiras descobertas, oferecendo tecnologia de ponta para tratar toda a gama de Síndromes Coronarianas Agudas.^5 , 6^ Nossa melhor compreensão da complexa dinâmica molecular desta doença é fundamental para melhorar ainda mais o diagnóstico precoce e prevenir seus resultados desastrosos.^7^

A medicina do século 21 oferece um grande arsenal de diagnósticos moleculares para várias doenças, mas a DAC está um pouco atrás.^8^

É amplamente conhecido que a inflamação desempenha um papel importante na aterogênese;^9^ assim, vários biomarcadores têm sido estudados nesse contexto, como a Proteína C-reativa, Interleucina (IL)6, Fator de Necrose Tumoral (TNF) alfa, entre outros.^10^ Um dos principais problemas dessas ferramentas é a falta de especificidade ou sua correta associação com a taxa de mortalidade.

Nesta edição, Tatlisu et al.,^13^ contemplam o uso de indutor fraco de apoptose semelhante ao fator de necrose tumoral solúvel (sTWEAK), um ator conhecido na proliferação e inflamação.^14^ para elucidar a calcificação da artéria coronária.

Em um estudo prospectivo, 139 pacientes consecutivos com Doença Renal Crônica (DRC) foram incluídos com mais angiografia coronária computadorizada (ACC) para análise do escore de cálcio da artéria coronária (CAC). Amostras de sangue foram coletadas para análise do sTWEAK, onde se buscou a relação com o escore CAC. Eles descobriram que, à medida que a pontuação CAC aumentava, os níveis de sTWEAK diminuíam significativamente. Além disso, eles concluíram que baixos níveis de sTWEAK foram um preditor de altos escores de CAC em sua população estudada.

É muito cedo para começar a adotar esses novos biomarcadores como padrão para o diagnóstico de DAC em nossa prática clínica; no entanto, esses tipos de esforços nos levam a uma visão aprimorada da dinâmica complexa do DAC, levando-nos ao futuro próximo da medicina de precisão, quando finalmente poderemos ter um ajuste na própria doença.
