# Supplementary material for: Devemos “Ajustar” Nossa Abordagem à Doença Arterial Coronariana?
Source: Arq Bras Cardiol. 2022 Aug 25;119(3):446–7. [Article in Portuguese] doi: 10.36660/abc.20220572 (PMC9438527; doi:10.36660/abc.20220572)
Supplement: Translation

# Translation: Should We “Tweak” Our Approach to Coronary Artery Disease?

**Authors:** Andres Felipe Valencia Rendón

**Affiliations:** 1 Brazil Universidade Federal do Rio de Janeiro, Rio de Janeiro, RJ – Brazil

**Keywords:** Coronary Artery Disease, Atherosclerosis, Cardiovascular Diseases/mortality, Biomarkers, Biomedical, Technology/trends

Despite our great advances in medical technology, Cardiovascular Disease remains the leading cause of death worldwide. Coronary Artery Disease (CAD) is one of the hallmarks.^1^

It is believed that the first correlation between coronary flow and angina pectoris was made by William Heberden in 1768, but its anatomical remarks were only described by 1829, when Jean Lobstein introduced the term Arteriosclerosis, reporting an abnormal formation in arterial walls.^2^ Later, in 1856, Rudolph Virchow suggested an arterial wall injury mechanism that could explain those findings from a cellular pathology compound.^3^ However, it was only until the 20^th^century, in the late 70´s, that the consortium of endothelial damage-wall shear stress-smooth muscle proliferation-thrombogenesis was first elucidated in the “Response to Injury Hypothesis” by Ross et al..^4^

Modern cardiology has evolved exponentially since those first discoveries, offering state-of-the-art technology to treat the full range of Acute Coronary Syndromes.^5 , 6^ Our better comprehension of this disease’s complex molecular dynamics is fundamental to improving an early diagnosis and further, preventing its disastrous outcomes.^7^

21^st^-century medicine offers a great armamentarium of molecular diagnoses for several diseases, but CAD is somewhat lacking behind.^8^

It is widely known that inflammation plays a major role in atherogenesis;^9^ thus, several biomarkers have been studied in this context, such as C-reactive Protein, Interleukin (IL)6, Tumor Necrosis Factor (TNF) alfa, among others.^10^ One of these tools’ mean issues is the lack of specificity or its right association with net mortality.

In this issue, Tatlisu et al.^13^ contemplate using soluble tumor necrosis factor-like weak inducer of apoptosis (sTWEAK), a known player in proliferation and inflammation,^14^ to elucidate coronary artery calcification.

In a prospective study, 139 consecutive patients with Chronic Kidney Disease (CKD) were included with further computed coronary angiography (CCA) for coronary artery calcium (CAC) score analysis. Blood samples were collected to analyze sTWEAK, where they sought the relationship with the CAC score. They found that as the CAC score increased, sTWEAK levels decreased significantly. Furthermore, they concluded that low sTWEAK levels were a predictor of high CAC scores in their studied population.

It is quite early to start adopting these new biomarkers as a standard for CAD diagnosis in our clinical practice; however, these kinds of efforts drive us to an enhanced view of the complex dynamic of CAD, walking us to the near future of precision medicine, when we could finally have a tweak to the disease itself.
